# Elevated free fatty acids in bulk tank milk: a dairy farm case report

**DOI:** 10.3389/fvets.2026.1778417

**Published:** 2026-03-23

**Authors:** Hannah M. Woodhouse, David F. Kelton

**Affiliations:** Department of Population Medicine, University of Guelph, Guelph, ON, Canada

**Keywords:** lipolysis, milk fat, milk quality, production, rancidity

## Abstract

While most fatty acids in milk are bound, some are in free form. Although free fatty acids (FFA) only represent approximately 0.1 % of total milk fat, they can have a large, detrimental impact on milk quality. Free fatty acids are products of milk fat lipolysis and begin at the farm level. Elevated concentrations of FFA in milk (≥1.2 mmol FFA/100 g of fat) can reduce milk foamability, impair cheese coagulation, decrease shelf life, and increase milk rancidity. By directly deteriorating the quality of milk, elevated FFA is a concern to the sustainability of dairy production that impacts all stakeholders. Since 2018, Dairy Farmers of Ontario (DFO) have tested FFA levels on every bulk tank milk sample, which is collected at every pick-up on all Ontario, Canada dairy farms. Results are reported to farmers, allowing for milk quality transparency and monitoring. In 2023, DFO field staff triggered an investigation of elevated FFA on a dairy farm with an average FFA of 1.32 ± 0.50 mmol/100 g of fat (between August 2018 and October 2022). The objective of this case report was to apply previously identified FFA risk factors in this on-farm investigation to identify farm-specific contributors to elevated FFA. The main FFA risk factors present on the farm were short and unequal milking intervals, poor milking equipment maintenance and sanitization, increased bulk tank milk agitation, and temperature fluctuations. High levels of fat supplements used in the lactating ration at certain times of the year may have also been a contributing factor. Elevated FFA concentrations are multifactorial, and increased awareness of farm-level risk factors through real-world examples can help farms implement management changes to improve milk quality.

## Introduction

Free fatty acids (FFA) result from milk fat lipolysis and are natural components of milk, but at extremely low levels (< 0.1% of milk fat) (1). Elevated concentrations of FFA in milk (≥1.2 mmol/100 g of fat) have become an increasing milk quality issue (2). Non-foaming of milk is one of the most common quality concerns associated with elevated FFA concentrations. Free fatty acids interfere with milk proteins that are responsible for stabilizing air bubbles during frothing, thereby reducing foam formation and stability (3). Free fatty acids also impair the protein binding and network formation required for effective cheese coagulation (4). In their free form, FFA can produce strong and unpleasant aromas and flavors that contribute to rancidity (5). When released from the triacylglycerol (TAG) molecule, FFA further destabilize milk fat, accelerating chemical reactions that drive flavor deterioration and reduced freshness (5).

Free fatty acid production begins on the dairy farm. Research suggests that FFA result from three main types of lipolysis: spontaneous, induced, and bacterial. Spontaneous lipolysis is catalyzed by lipoprotein lipase (LPL), and factors contributing to spontaneous lipolysis occur before milk harvest (5–7). Induced lipolysis is caused by physical damage to the milk fat globule membrane, and this type of lipolysis occurs during and after milk harvest (8, 9). Bacterial lipolysis is catalyzed by bacterial enzymes (such as those from psychotropic bacteria) and occurs exponentially during milk storage, especially in warmer or contaminated environments (10). A time temperature recorder (TTR) is a tool used on farms to help to inform producers when milk or wash temperatures are not met.

Elevated levels of FFA in bulk tank milk affect everyone: farmers (who in some regions are not paid for their milk shipment), processors (who cannot create a high-quality dairy product), retailers (for example, coffee shops, who have to deal with non-foaming milk), and consumers (who are dissatisfied with their purchased high FFA dairy product). Because of the numerous consequences of elevated FFA, it is an important milk quality attribute to monitor.

In Ontario, Canada, there are over 3,000 farms with over 50 % still milking in tie stall facilities (11). Since August 2018, the Dairy Farmers of Ontario (DFO) milk marketing board has included FFA in their bulk tank milk quality tests. Farmers receive their bulk tank milk FFA results with their other milk components. Free fatty acid levels are reported in mmol/100 g of fat and classified as either “elevated” (≥1.2 mmol/100 g of fat) or “normal” (< 1.2 mmol/100 g of fat). There is currently no penalty structure for elevated FFA in Canada, but farmers risk the milk test being traced back to their farm (and therefore receiving a quality penalty) if complaints are brought forward by processors or retailers.

Due to Canada's supply-managed dairy system, one farm's high FFA milk can have a significant negative impact because milk is a pooled product. This can be detrimental to retailer and consumer satisfaction, which is what drives domestic milk markets and incentives (increased allotment of dairy quotas). Therefore, there are many reasons why it is an economical decision for farmers to pay attention to their FFA levels, identify their farm's FFA risk factors, and act when appropriate.

Research in Canada and Europe has demonstrated that FFA are multifactorial, and a combination of risk factors from all forms of lipolysis can result in an elevated bulk tank FFA test (9, 12). Although numerous observational studies have identified risk factors for elevated FFA, there are few published examples demonstrating how this knowledge can be translated into practical on-farm diagnostics. The objective of this case report was to apply previously identified FFA risk factors in an on-farm investigation to identify farm-specific contributors to elevated FFA concentrations. This approach demonstrates how epidemiologic evidence can be translated into practical on-farm diagnostics to mitigate FFA and improve milk quality.

## Case description

This case report involves a singular Ontario tie stall dairy farm that milked approximately 40 Holstein cows. In January 2023, DFO field staff triggered an investigation of elevated FFA (≥1.2 mmol/100 g of fat) on this farm due to milk quality concerns. The farm signed a consent form to collect data as part of another research project that was approved by the Research Ethics Board (REB) of the University of Guelph (REB no. 22-07-031). All data were handled in a manner that preserved farm confidentiality.

All farm-specific historical bulk tank data between August 1, 2018 and October 22, 2022 were obtained from DFO and examined. Results after October 22, 2022 were not available at the time of the investigation. All bulk tank sample tests were conducted at the University of Guelph Food Laboratory (Guelph, ON, Canada). The compositional data of interest included FFA (mmol/100 g of fat), milk fat (% weight/volume), milk protein (% weight/volume), SCC (thousand cells/mL), and milk shipment yield (L). Bacteria levels measured weekly by BactoScan (thousand bacteria/mL) were also reviewed. All milk component data of interest that were collected are reported in Table 1.

**Table 1:** Milk component results from every-other-day milk pick-ups between August 1, 2018 and October 22, 2022.

**Variable**	***n***	**Mean**	**SD**	**Min**	**Max**
Milk (L)	752	2999.42	316.17	1,320	3,909
Fat (% weight/volume)	752	4.09	0.17	3.57	4.73
Protein (% weight/volume)	752	3.02	0.08	2.74	3.23
SCC (thousand cells/mL)	752	202.52	77.80	64	495
Bactoscan^a^ (thousand bacteria/mL)	221	14.41	21.30	2	200
FFA (mmol/100 g of fat)	752	1.32	0.50	< 0.01	4.67

^a^Bactoscan results were less frequent because they were only tested weekly.

**Figure 1:**
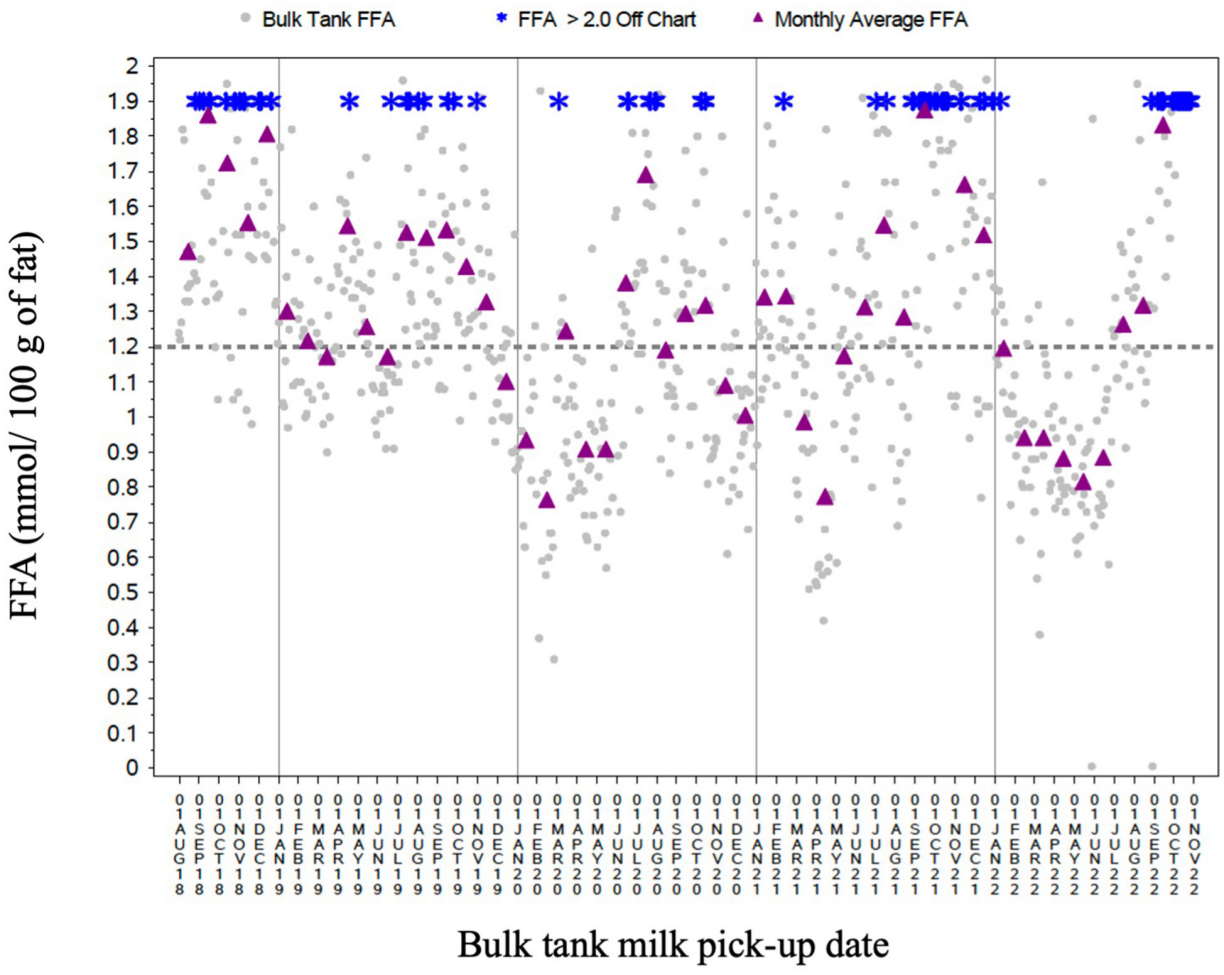
Scatterplot of this farm's bulk tank FFA test results between August 1, 2018 and October 22, 2022.

There were 752 bulk tank FFA samples from this farm recorded. Bulk tank pick up FFA results were reported as high (≥1.20 mmol/100 g of fat) or normal (< 1.20 mmol/100 g of fat). Over 54 % (*n* = 413) of samples had elevated FFA. The mean FFA concentration was 1.32 ± 0.50 mmol/100 g of fat. Monthly average FFA levels were computed, and both pick-up values and monthly values were graphed (Figure 1). The graph illustrated high FFA levels throughout the testing time period, with some yearly and seasonal differences.

After gathering these preliminary data, the farm was visited for observation and further data collection. Farm information related to the herd, lactating ration, milking system, and milk storage was collected using a Qualtrics XM platform (https://www.qualtrics.com) survey. Time temperature recorder data for the bulk tank were extracted by ejecting the TTR's memory card and inserting it into a computer to transfer the TTR files (specific details on data retrieval can be found here: http://dairycheq.com/files/Users_manual_EN-FR_A5.pdf). The TTR files (in .DAT format) were saved to the computer and viewed using Dairy Cheq software to observe in both a graphical and list format.

## Diagnostic on-farm assessment

This was an Ontario conventional tie stall farm milking approximately 40 Holstein cows, three times a day. Time temperature recorder data were extracted, and milking times were identified (identifying “vacuum on” and “vacuum off” time-stamped signals). There were noticeable time interval differences between milkings. The morning milkings started between 6:00 and 9:00 a.m., afternoon milkings were between 12:00 and 3:00 p.m., and evening milkings were between 8:00 and 11:00 p.m. The milking times were very inconsistent, especially in the mornings. The shortest intervals were between the morning and afternoon milkings. The TTR data indicated that some of the bulk tank milk pick-ups were very close to the afternoon milking time. Bulk tank washes were performed immediately after milk pickup, but pipeline washes were sometimes missed or started more than an hour after the morning milking. Wash temperatures, indicated by the TTR, were over 50 °C most of the time, but some TTR readings had a peak wash temperature in the low 40 °C range. Cold acid rinses after a bulk tank wash were not performed.

The milk temperature in the bulk tank was consistently around 3 °C. The tank was top loading (i.e., milk entered from above), and the temperature probe was located at the back of the bulk tank. The tank appeared to be adequate in size for the farm. The bulk tank was equipped with a single agitator, which ran for seven min every hour. A single-stage plate cooler in the milk house was present, and it appeared to work efficiently, though the farmer mentioned that it is less efficient on hot summer days. It was noted that the milk filter gets changed at every milking (three times a day).

**Figure 2:**
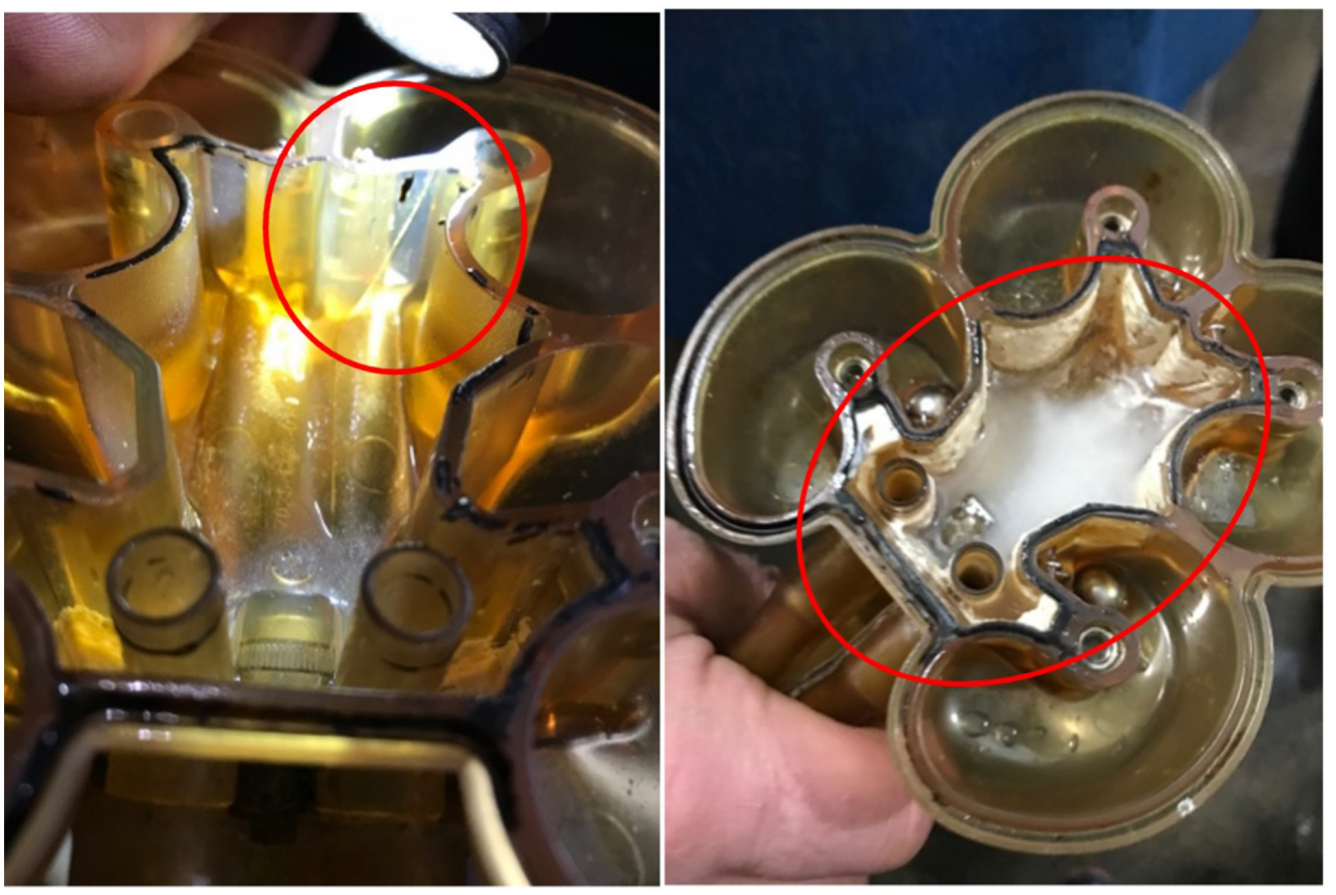
Cracks and milk seepage in the GEA IQ model milking units on this farm.

The milking machine units (GEA IQ automatic take-off model) had noticeable cracks in the claws of one-third of the units, as well as gasket leaks. The units were dismantled to discover evident signs of milk seepage and curdled milk (Figure 2). An airflow analysis was conducted using these units, and the results showed high levels of vacuum fluctuations in the damaged units. The vacuum in the pipelines was noted to be 15.2 mmHg. The tie stall milking system had overhead pipelines that were 2 inches in diameter in the barn, and 1.5 inches in diameter in the milk house. Pipeline length and number of turns from the cow (milking unit) to the bulk tank (bottom of tank) were noted and measured (Table 2). The farmer mentioned that newer pipelines were installed in 2017, including some with a “T” shape. Some of the “T” shaped pipelines were dismantled and inspected, and there were noticeable signs of debris.

**Table 2:** Barn pipeline diameter, length, number of turns and elevated sections.

**Barn pipeline diameter (inches)**	**Milkhouse pipeline diameter (inches)**	Average horizontal pipeline distance (ft)^*^	**Overall vertical pipeline distance (ft)**	**Maximum pipeline height (ft)**	**Number of pipeline turns**
2	1.5	103.2	20.2	5.8	22

^*^This is the average distance of the furthest and closest cow to the bulk tank.

The herd appeared to be in good body condition and care. The lactating ration was assessed, and the farm's nutritionist was consulted. Palm fat was fed at a level of 300 g per head/day, but it was noted that higher levels are fed during incentive days (normally during the fall months, when farmers are permitted to ship more milk). There were no other concerns with the ration quality or quantity.

## Discussion

This was a case of a high FFA farm with many potential risk factors based on previous research (9, 12). This farm's FFA average (1.32 mmol/100 g of fat) far exceeded the Ontario industry average of 0.83 mmol/100 g of fat (13). Although this average is below the milk sensory threshold of 1.20 mmol/100 g of fat, over 54% of the samples were classified as elevated, demonstrating that there are both permanent and changeable FFA risk factors present on the farm. However, it is important to note that data collection occurred at a single point in time, which could influence observations reported.

Figure 1 illustrates the impact that the time of year had on FFA. Free fatty acid levels appeared lowest in the spring months and highest in the fall months, which is consistent with the literature (13). There was also greater FFA variability and more occurrences of extremely high FFA results (>2.0 mmol/100 g of fat) in the fall months. Efforts from the farm to increase butterfat production during fall incentives (increased quota allotment that would allow farmers to ship more milk) could be a contributing factor here. At the time of the visit, the farm was feeding a fat supplement to support milk fat production. However, despite January 2023 having no incentive days, the fat supplement dose of 300 g/head/day was on the high end of what is recommended (14). The farmer noted that higher doses of fat supplements are used during incentive days, and this could also contribute to the rise in FFA during the fall months. A previous study identified an association between elevated FFA and times of the year when there are three or more incentive days in a month (15). Fat supplements (such as palm fat) created enlarged milk fat globules that are fragile and prone to lipolysis (16). Another strategy used to fill quota during incentive days is to extend the lactation of cows. However, late lactation cows have higher levels of LPL that can contribute to more spontaneous lipolysis (17). This research did not examine the individual cow characteristics, such as DIM, although this would have been beneficial.

Insufficient cleaning concerns identified by the TTR could contribute to bacterial lipolysis. Delayed pipeline washes and lower wash temperatures can increase bacteria levels that contribute to lipolysis. The delayed pipeline wash might have also contributed to the residual milk in some pipeline sections (i.e., “T” section where milk can more easily get trapped) that would cause bacterial build-up over time. There was also residual milk with evident signs of bacterial growth in the milking machine units. This could be due to delayed or insufficient cleaning, but another contributing factor is the neglected maintenance on these machines.

The cracks not only allowed for milk seepage but also contributed to higher vacuum levels and fluctuations, which can increase FFA through induced lipolysis. Multiple studies have demonstrated a relationship between factors that contribute to more milk fat globule contact and increased FFA (8, 9, 12). These milking units also had individual air inlets for each quarter, which can contribute to increased vacuum fluctuations and FFA as well (8, 9). However, the milking machines were automatic take-offs, which prevented overmilking (another risk factor for FFA) (18). This is likely a protective measure for this farm due to its high milking frequency and unequal milking intervals.

The unequal milking intervals, especially between the morning and afternoon milking, were concerning for FFA and likely contributed to spontaneous lipolysis. Multiple studies have demonstrated the relationship between higher milking frequencies or shorter milking intervals and increased FFA (12, 18–20). This is partially due to insufficient time for milk fat globule membrane formation that protects it from induced lipolysis and a higher proportion of large milk fat globules (9, 18). Milking three times a day is less common in tie stall systems compared to automated milking systems due to labor requirements, but both systems are associated with higher FFA levels compared to parlor milking systems (9, 12, 21). In a previous study that investigated farm factors associated with FFA on Canadian dairy farms, tie stall milking systems milking three times a day were associated with a 1.30 mmol/100 g of fat increase in FFA compared to parlor farms milking three times a day (9).

Another risk factor for high FFA levels in tie stall milking systems compared to parlor milking systems is pipeline configuration. Higher pipelines with more turns and elevated sections can put mechanical stress on the milk fat globules as they travel from the cow to the bulk tank, due to increased air admission demands (8). The pipeline vacuum levels and lengths were comparable to other tie stall farms, but still greater than the average in parlor milking facilities, which could contribute to lipolysis (9).

The presence of a plate cooler on the farm was likely protective against FFA production. Studies have indicated that pre-cooling can help to reduce FFA levels by as much as 0.25 mmol/100 g of fat (9, 12, 22). Cooler milk entering the bulk tank can prevent bacterial growth and thus lipolysis (23). Induced lipolysis rates are also lower due to the bulk tank milk cooler not having to run as immediately, or for that long, therefore reducing agitation of milk (8). However, this farm's agitation length of seven min every hour was quite long for the small bulk tank size and could be reduced to minimize milk disturbance and maintain milk fat globule structure (24).

A plate cooler can also help prevent temperature fluctuations in the bulk tank, which is another stressor that can lead to FFA production (19, 22). Pre-cooling milk can reduce the risk of milk freezing and high FFA levels. A previous study by Woodhouse et al. (25) demonstrated a 0.36 mmol/100 g of fat increase in FFA when a “milk too cold” TTR alarm (which indicated milk freezing) was present. Although there were minimal “milk too cold” TTR alarms and a pre-cooling mechanism was in place on the farm, there were still temperature fluctuations that occurred. The bulk tank milk was often washed immediately before the afternoon milking, and cold milk entering a hot tank could contribute to FFA (22). A cold acid rinse before the start of milking could further reduce lipolysis.

## Conclusion

This case report applied previously identified FFA risk factors in an on-farm investigation to identify farm-specific contributors to elevated FFA. It also outlined the protective factors against FFA. The main FFA risk factors present on the farm were short and unequal milking intervals, poor milking equipment maintenance and sanitization, increased bulk tank milk agitation, and temperature fluctuations. High levels of fat supplements used in the lactating ration at certain times of the year may have also contributed to elevated FFA levels. This farm illustrates how multiple, interacting factors related to lipolysis can drive elevated FFA concentrations. Addressing milking equipment issues may be a practical starting point to reduce FFA, while equalizing milking intervals is another low-cost management strategy. Overall, this case report demonstrates how epidemiologic evidence can be translated into practical on-farm diagnostics to mitigate FFA and improve milk quality.

## Data Availability

The original contributions presented in the study are included in the article/supplementary material, further inquiries can be directed to the corresponding author.
